# Machine Learning in Mammals’ Health: Integrating Multi-Omics Data for Precision Nutrition and Sustainable Production

**DOI:** 10.3390/vetsci13101122

**Published:** 2026-10-21

**Authors:** Weihao Huang, Xudong Yu, Yaoyao Xia, Weijian Li, Xuan Zhao

**Affiliations:** Animal Nutrition and Bio-Feed, Key Laboratory of Chongqing Education Commission of China, Southwest University, Chongqing 400715, China; huang-weihao@foxmail.com (W.H.);

**Keywords:** machine learning, precision nutrition, intestinal microbiota, biomarker identification, intestinal health

## Abstract

Precision nutrition in modern mammalian production aims to transition from static feeding regimens toward dynamic and customized strategies by unraveling complex interactions among genetics, environment, the gut microbiome, and host physiology. Machine learning provides a powerful analytical framework to navigate high-dimensional multi-omics datasets. However, existing applications frequently lack systematic criteria for algorithm selection and remain largely confined to observational statistical associations without progressing toward causal verification. This perspective delineates a coherent translational chain spanning three interconnected operational scenarios: key functional microbiota screening to identify whom to intervene on, multi-omics biomarker identification to define when to intervene, and host pathway characterization to reveal how the animal responds. We critically examine the operational boundaries, interpretability trade-offs, and failure modes of classical algorithms including LASSO, support vector machines, random forest, and graph neural networks across these domains. Furthermore, we address critical field bottlenecks such as high dimensionality, small-sample overfitting, batch effects, and black-box opacity, while highlighting emerging paradigms including multimodal data fusion, transfer learning, and causal machine learning to bridge the gap between data-driven screening and actionable nutritional interventions. Ultimately, transforming machine learning from an auxiliary predictive tool into an autonomous precision nutrition engine requires shifting the research focus from isolated model accuracy to reproducible and causally validated biological targets.

## Simple Summary

Modern livestock farming is moving away from traditional, uniform feeding methods toward precision nutrition, which tailors diets to the specific biological needs of individual animals. Understanding the complex dialogue between an animal, its feed, and its gut microbiome requires examining multiple biological layers, including genetic traits, intestinal bacteria, and metabolic products. While these detailed datasets are challenging to analyze, advanced computational techniques such as machine learning can uncover hidden patterns, help screen beneficial gut bacteria, discover early warning biomarkers for health issues, and clarify how animals absorb nutrients. However, many current computer models only identify statistical correlations without confirming true biological causes, and they often struggle with small sample sizes and limited transparency. This paper reviews how computational methods can be purposefully selected to evaluate animal health and digestive pathways, address data limitations, and move beyond simple associations toward confirmed cause-and-effect discoveries. Ultimately, establishing reliable computational frameworks will help livestock producers design targeted feeding strategies, improve feed efficiency, reduce environmental waste, and promote sustainable animal welfare.

## 1. Introduction

The modern animal production industry faces intertwined challenges of resource utilization efficiency, environmental governance, and economic feasibility. Animal health and productive lifespan are profoundly influenced by multifactorial interactions among genetics, environment, and complex gut microbiota [1]. To overcome the constraints of conventional herd-level management, precision nutrition has emerged as a cornerstone paradigm for sustainable animal production. Precision nutrition can be defined as the practice of tailoring dynamic, individual- or group-specific nutrient delivery in real time based on the animal’s genetic background, physiological stage, metabolic state, gut microbiome composition, and environmental exposure, thereby maximizing feed conversion efficiency, safeguarding health, and minimizing environmental footprints [2]. However, implementing precision nutrition requires continuous, high-resolution deconvolution of the intricate dialogue within the “feed–microbiome–host” axis, a task that inherently generates high-dimensional, heterogeneous multi-omics data. This analytical bottleneck necessitates the adoption of Machine Learning (ML). Unlike static statistical formulations, ML provides an adaptive computational engine capable of learning complex nonlinear relationships, extracting high-order feature representations, and uncovering latent patterns across multi-omics modalities, thereby serving as an indispensable tool to translate multidimensional biological data into actionable nutritional strategies [3].

Recent advancements across functional omics have begun to establish the biological groundwork for this precision framework [4,5]. Specifically, notable progress has been made in screening functional microbial communities (such as characterizing the protective role of specific taxa like *Lactobacillus* in maintaining mucosal barrier integrity and counteracting intestinal inflammation) [6], identifying metabolic biomarkers (such as capturing discriminative lipid and metabolite signatures, including 3-hydroxybutyric acid and 9-oxo-ODE, that sensitively signal systemic metabolic shifts) [7], and characterizing host functional pathways (such as mapping the spatial distribution of intestinal nutrient transporters along the villus axis to guide targeted additive release, or screening minimal host gene expression signatures to monitor physiological recovery) [8,9]. Nevertheless, harnessing these biological discoveries for real-world precision feeding remains hindered by a fundamental computational bottleneck: high-dimensional multi-omics data are inherently characterized by multi-way combinatorial effects and noise, which conventional univariate or linear statistical analyses fail to disentangle, frequently falling short in separating true causal drivers from passenger statistical correlations. ML directly bridges this divide by orchestrating systematic biomarker discovery and dynamic predictive modeling [10]. However, formulating these targeted intervention strategies first requires identifying the specific functional bacterial taxa responsible for conferring health benefits or driving disease phenotypes from highly diverse and complex microbial communities.

To systematically integrate multi-omics in precision nutrition, it is critical to delineate the primary omics technologies, their distinct data characteristics, and the specific biological information tiers they provide (Table 1). In mammalian nutrition, multi-omics captures a hierarchical molecular continuum spanning from static predisposition to functional endpoints. Specifically, genomics deciphers static individual genetic architectures and quantitative trait loci (QTLs) underlying feed efficiency, dictating what could happen across an animal’s lifespan. Transcriptomics captures dynamic intermediate mRNA expressions and alternative splicing events across tissues, revealing what is triggered to happen in response to dietary transitions or stressors. Proteomics quantifies functional digestive enzymes, nutrient transporters, and mucosal barrier proteins along the intestinal tract, directly answering what makes it happen. Downstream, metabolomics profiles dynamic low-molecular-weight metabolites, including circulating short-chain fatty acids (SCFAs) and lipid species, providing functional readouts of what has happened regarding real-time metabolic and energy fluxes. Complementing host measurements, microbiomics and metagenomics (16S rRNA and shotgun sequencing) characterize luminal microbial diversity, CAZymes, and functional metabolic pathways, identifying who is there and what they can do to digest structural feeds and modulate gut barrier integrity. Integrating these multi-layered modalities enables computational frameworks to trace how dietary ingredients are transformed by microbiota, how microbial cues signal to host receptors, and how host tissues functionally respond (Table 1).

However, two notable research gaps remain. First, most studies merely enumerate the algorithms employed without systematically comparing the scenarios in which different algorithms are more suitable. When is Least absolute shrinkage and selection operator (LASSO) preferable to Random Forest (RF)? Under what conditions does Support Vector Machine (SVM) outperform Extreme Gradient Boosting (XGBoost)? Such methodological questions have rarely been addressed in existing reviews [15]. Second, current research largely stops at statistical associations, with insufficient discussion on how to progress from association to causal verification and subsequently to actionable nutritional intervention strategies. This perspective aims to address these two gaps by comparing the applicability boundaries of different methods across three major application scenarios and analyzing the shared bottlenecks facing the field.

## 2. Machine Learning-Assisted Screening of Key Functional Bacteria

The gut microbiota participates in food digestion and absorption, and protects host health by maintaining barrier integrity, competitively inhibiting pathogen colonization, and modulating the immune system [14]. In intensive animal production, environmental stressors, dietary transitions, or pathogen challenges frequently perturb this symbiotic microbial equilibrium, precipitating gut dysbiosis, compromised epithelial barrier function, and subsequent intestinal inflammation [2,6]. Experimental colitis models offer a quintessential pathological paradigm to interrogate these gut dysbiosis-driven inflammatory cascades, which arise from aberrant interactions among host genetics, external environmental triggers, and the collapsed microbiota [10]. Within the precision nutrition framework, targeted microbial interventions, most notably the precise supplementation of functional probiotics or postbiotics, represent an effective strategy to counter such intestinal inflammation, re-establish microbial eubiosis, and accelerate mucosal healing. Screening for key functional bacteria is a prerequisite for developing targeted intervention strategies, and ML can efficiently identify reproducible microbial features associated with health or disease states by analyzing and modeling multiple microbiota datasets [5].

Guo et al. [6] employed L1-regularized logistic regression to identify the *Lactobacillus* genus, whose abundance was reduced in a colitis animal model. The strength of this approach lies in its automatic feature selection and coefficient interpretability. The bacterial genus weights can be directly interpreted as protective or risk factors. However, the linear assumption of LASSO is its core limitation. It may overlook functional microbial groups that exhibit nonlinear synergistic interactions. In contrast, tree-based models such as random forest can capture nonlinear relationships and output feature importance rankings, but these rankings cannot directly indicate the direction of biological effects, and such models are more prone to overfitting under small-sample conditions [16,17]. This methodological divergence highlights an important issue: algorithm selection depends on research objectives. If the goal is to identify interpretable candidate targets for subsequent validation, LASSO is more appropriate; if the goal is to maximize prediction accuracy by identifying complex microbial marker combinations, random forest may be preferable. Yet this selection logic is rarely explicitly discussed in published studies. ML can integrate multiple data features [4] and combine data from multiple sources to reveal interactions between microorganisms and the host or environment [18], which opens the possibility of multi-algorithm joint application.

A more fundamental limitation of current research is that most microbial screening studies establish statistical associations rather than causal relationships. Designing probiotic intervention strategies based on associations that have not undergone causal validation may lead to failure in subsequent verification experiments. Therefore, introducing Mendelian randomization or causal graph models into the screening workflow to distinguish causal driver bacteria from companion bacteria is a key direction for improving target reliability.

## 3. Machine Learning Techniques for Biomarker Identification

Metabolites of the gut microbiota play a key role in both normal physiological states and disease development [12]. Biomarker discovery typically requires feature selection from high-dimensional metabolomic data. Researchers often combine multiple ML algorithms to improve reliability. For instance, LASSO, SVM, and random forest can be jointly applied, with the intersection of their results serving as potential biomarkers, using inter-method consensus as a proxy for reliability [11]. However, it is critical to recognize that consensus across multiple algorithmic architectures on the same dataset does not confirm biological reliability, as different algorithms may still converge on shared dataset-specific biases, batch effects, or spurious statistical correlations. Rather than providing definitive biological ground truth, such consensus across methods serves primarily as an efficient prioritization heuristic and hypothesis-generating filter to narrow down the vast multi-omics feature space. Ultimately, these computational candidates are meant to provide directional guidance and manageable candidate targets for subsequent targeted wet-lab experimental validation, independent cohort replication, and causal perturbation. Subsequent integration with single-cell sequencing and transcriptomics data can further reveal the pathways that these biomarkers may regulate.

When screening key biomolecules from databases, researchers may face massive data volumes that impose high computational costs. ML can learn data features and reduce dimensionality [15]. Luttens et al. [19] employed virtual screening with CatBoost classifiers and graph neural networks to pre-screen compounds, performing molecular docking only on those predicted as highly active, thereby reducing computational costs to one-thousandth of traditional methods. This coarse-to-fine screening strategy offers a useful reference for dimensionality reduction in large-scale metabolomic data.

Distinct differences exist in the applicability conditions of different algorithms, yet existing reviews rarely compare them systematically. LASSO regression is suitable when the number of features far exceeds the sample size and a sparse biomarker set is desired. Its strength lies in directly providing linear coefficients, with each selected feature having a clear biological effect direction, facilitating subsequent experimental validation and the applicable scenario is screening a set of interpretable, individually verifiable core biomarkers. SVM handles nonlinear boundaries through kernel functions and performs well with small-sample, high-dimensional data. In animal model studies, SVM has played a pivotal role in distinguishing disease groups from controls using complex serum proteomic features and can improve diagnostic accuracy by analyzing combinations of multiple biomarkers [11,14,20]. However, SVM cannot easily quantify the contribution of individual features, its applicable scenario is high-accuracy classification rather than biological interpretation of each biomarker’s contribution [21].

In the context of multi-omics biomarker identification, random forest serves as a robust ensemble learning architecture capable of evaluating high-dimensional feature spaces and ranking candidate signatures based on decision-tree importance metrics [11,22,23]. For instance, Tan et al. [7] utilized machine learning classifiers including random forest, support vector machines, and logistic regression. To characterize gut microbiota, serum metabolome, and lipidome alterations in individuals with type 1 diabetes. By applying these algorithms across different taxonomic tiers and molecular profiles, their models effectively screened out discriminative multi-omics candidate biomarkers (such as key gut taxa and serum metabolites including 3-hydroxybutyric acid and 9-oxo-ODE), which were subsequently validated in an independent cohort to confirm their diagnostic relevance and disease associations. Despite these advantages, random forest possesses several notable limitations. For example, while individual decision trees are human-readable, aggregating large forests obscures straightforward biological interpretability, and the model is generally less suited for fine-grained continuous regression than for classification tasks. Furthermore, in small-sample high-dimensional omics datasets, random forest remains susceptible to overfitting and fitting spurious correlations, and its calculated feature importance scores reflect observational associations rather than true causal relationships [23,24].

In practice, a serial strategy of “dimensionality reduction first (LASSO pre-screening), ensemble modeling second (RF modeling), and independent validation last” can help balance interpretability and prediction accuracy. Furthermore, ML models can assess feature importance for prediction outcomes, thereby revealing which features are key factors in distinguishing sample groups [4], which is valuable for understanding disease mechanisms and designing intervention strategies.

## 4. Exploration of Host-Regulated Pathways

The acquisition of host data benefits from ML techniques. Spatial proteomics requires tissue sectioning and regional LC-MS/MS analysis. Hu et al. [8] developed the Plato framework, which integrates Flow2Spatial technology for whole-tissue-level reconstruction, reduces the dimensionality of 2D spatial data, and employs deep learning with transfer learning using H&E staining or spatial transcriptomics data as the training set, ultimately establishing a high-precision spatial proteomic map of intestinal villi. In the context of livestock precision nutrition, these findings offer a transformative blueprint for resolving regional digestive and absorption heterogeneity along the mammalian gastrointestinal tract. In commercial swine and cattle production, different intestinal segments exhibit vastly distinct digestive microenvironments and transporter landscapes. By pinpointing the high-resolution spatial distribution of region-specific peptide, amino acid, and mineral transporters along the villus axis, this computational framework directly informs the spatial formulation of livestock rations. Specifically, it guides the design of targeted-release feed additives and microencapsulated functional amino acids engineered to release payloads at specific intestinal coordinates, thereby maximizing site-specific nutrient uptake and curtailing unabsorbed nutrient excretion.

In host transcriptomic analysis, Adriaensen et al. [9] employed random forest with leave-one-out cross-validation to screen extensive gene expression data, identifying four differentially expressed genes (PRSS33, IL10, SLFN14, and HRH4) as surrogate cure markers, with model stability and generalizability validated through 1000 bootstrap iterations. This methodology directly addresses the high operational cost and complexity of livestock herd monitoring. High-throughput sequencing of entire transcriptomes is cost-prohibitive for routine farm-level diagnostics in livestock herds. However, the core principle of using ML to compress complex physiological states into a minimal, representative host gene panel enables cost-effective herd health management. In practical livestock production, such minimal gene panels could be deployed via rapid, low-cost RT-qPCR assays using peripheral blood or mucosal scrapings to continuously track whether cattle or pigs have recovered from metabolic stress, dietary weaning shock, or subclinical enteritis following targeted nutritional interventions, thereby operationalizing dynamic feed adjustment in commercial settings.

**Figure 1:**
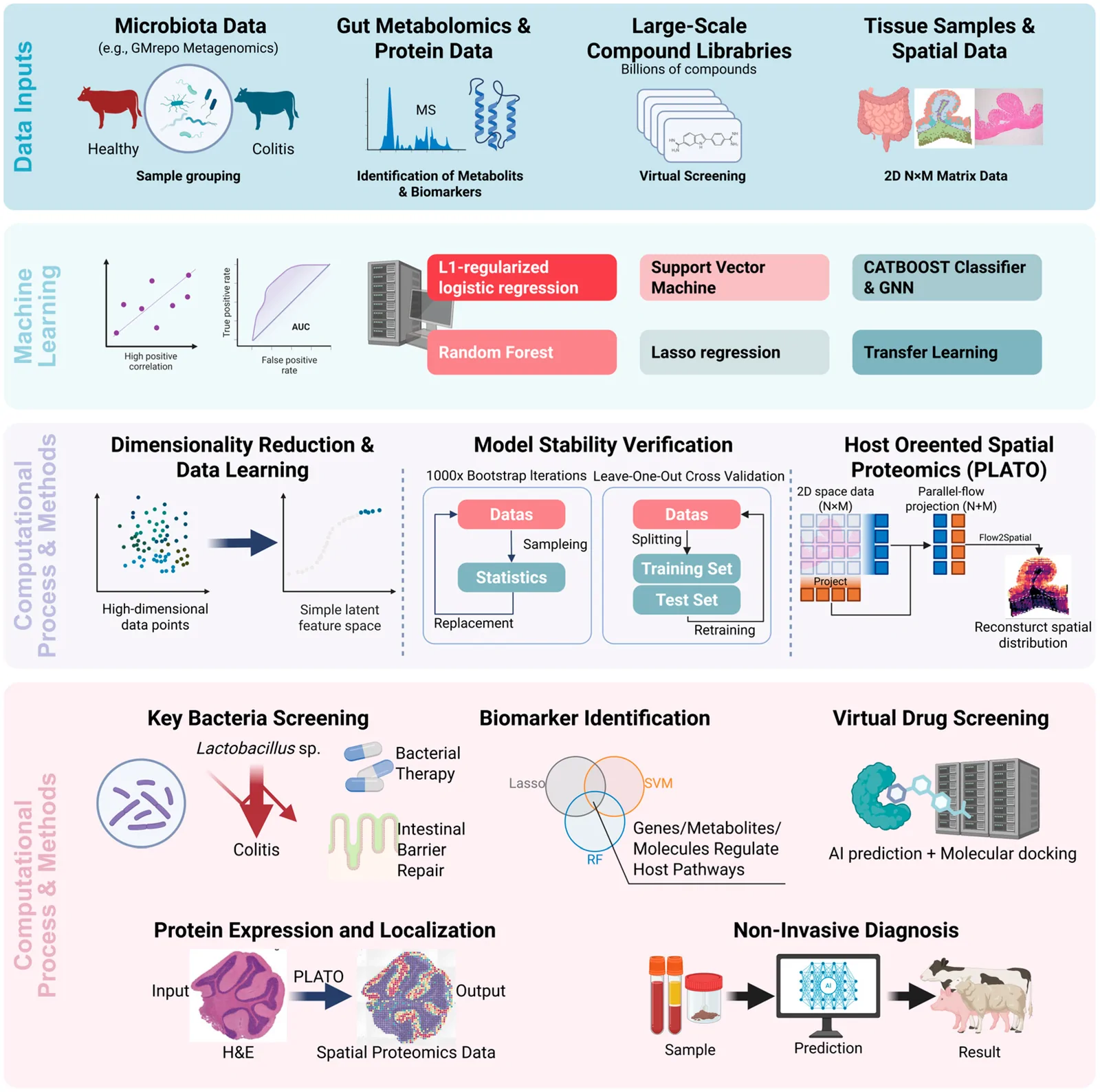
Data Sources, Workflow, and Applications of Machine Learning. (Created in bioRender. Weihao, H. (2026) https://app.biorender.com/illustrations/69a65131eae6de59437f2adb?slideId=f95737a3-753c-4e72-8dbc-bc351b08b2c8, accessed on 12 October 2026).

In the analysis of host diseases, ML holds the promise of replacing traditional invasive diagnostic methods. By utilizing data from previous animal experiments, ML can extract latent features from phenotypic characteristics and blood samples, reducing animal discomfort and risk while improving diagnostic accuracy and convenience (Figure 1) [25]. In intensive livestock production, minimizing invasive procedures such as tissue biopsies or liver aspirations is critical for preserving animal welfare and preventing stress-induced productivity losses. Deploying ML models that interpret non-invasive or minimally invasive inputs enables continuous, stress-free surveillance of subclinical systemic disorders in farm animals, ensuring timely nutritional recalibration without compromising herd performance.

Thus, the three application scenarios are interconnected within the precision nutrition framework. Key microbial screening answers “whom to intervene on,” biomarker identification answers “when to intervene,” and host pathway characterization answers “how the animal responds after intervention.” Together, they form a research chain from target discovery to efficacy verification.

The relationships among these three scenarios are illustrated in Figure 1. In this workflow, the data layer integrates multi-modal data including metagenomics, metabolomics, and host transcriptomics/proteomics, where batch effects from different data sources must be addressed as a priority. The feature layer selects algorithms according to research objectives. LASSO is preferred when the goal is screening interpretable targets, whereas SVM or random forest should be considered for complex classification tasks and achieves cross-omics feature fusion through dimensionality reduction [21,24]. The core challenge of the model layer lies in controlling overfitting under small-sample conditions, making cross-validation and independent validation sets essential. The decision layer translates model outputs into verifiable biological hypotheses or nutritional intervention strategies, with subsequent experimental or production data feeding back into model iteration. The central premise of this framework is that the value of ML in precision nutrition should be measured not solely by model accuracy, but by whether its outputs can be translated into implementable intervention strategies.

It is worth noting that the work of Hu et al. [8] reveals that nutritional intervention can be not only about “what to supplement” but also “where to supplement”. For instance, delivering functional amino acids to specific intestinal segments via targeted-release technologies. The strategy demonstrated by Adriaensen et al. [9], achieving complex physiological state discrimination with only four genes, suggests that nutritional intervention effects could potentially be dynamically assessed through monitoring the expression of a small number of host genes, substantially lowering monitoring costs and making precision nutrition more feasible in production settings (Figure 1).

## 5. Current Limitations and Future Directions

A systematic comparison of representative machine learning models, highlighting their primary biological utilities and methodological limitations across diverse omics and biomedical tasks, is summarized in Table 2. Understanding these task-specific trade-offs between predictive capacity and interpretability is essential for guiding model selection and optimizing computational workflows. The application of ML in mammalian precision nutrition still faces several bottlenecks.

Small sample sizes and data heterogeneity. Animal experiments are costly and omics testing is expensive, resulting in sample sizes often far smaller than the number of features. Models are prone to overfitting-exhibiting strong discriminative ability in retrospective data but deficient performance in predictive data [54]. Batch effects arising from different farms, breeds, and dietary conditions severely compromise model generalizability. In metabolite research, spatial isomers are often not distinguished, leading to loss of structural diversity data.

Insufficient interpretability. The decision-making processes of complex ensemble models such as deep learning and XGBoost are difficult to interpret. In biomedical research where rigorous mechanistic explanation is required, this undermines researcher confidence. Consequently, ML is currently often confined to assisting in the screening process [31].

To address these shortcomings, multimodal data fusion can enhance model reliability by integrating data from genomics, transcriptomics, proteomics, metabolomics, and microbiomics to construct a more comprehensive molecular map of disease pathogenesis [55]. Furthermore, precision nutrition in livestock inherently requires continuous, longitudinal monitoring rather than static cross-sectional snapshots. Sequence architectures like Long Short-Term Memory (LSTM) networks excel at deciphering time-dependent nutritional phenotypes. For instance, Blake et al. [45] demonstrated that an LSTM sequence model can accurately and generalizably predict individual daily dry matter intake in grazing beef cattle from pragmatic longitudinal data, substantially outperforming standard static nutritional equations and illustrating the potential of dynamic sequence modeling for scalable feed-efficiency evaluation in livestock. Transfer learning offers a powerful paradigm to bridge data-scarce downstream tasks and large-scale upstream reference profiles, effectively mitigating the small-sample dilemma [51]. By transferring representations learned from data-rich reference modalities such as histological images, spatial transcriptomics, or bulk omics to target cohorts with limited measurements, transfer learning enables cross-modal pattern reconstruction and robust feature extraction in low-data regimes. For instance, cross-omics transfer learning frameworks such as Flow2Spatial leverage generative architectures and shared latent spaces to infer hard-to-measure molecular distributions from accessible reference omics, demonstrating how pre-trained latent structures can enhance generalization across diverse biological tasks. Causal ML, which combines traditional causal inference methods, can improve model interpretability, though it must still be applied with caution in clinical diagnosis [56].

**Figure 2:**
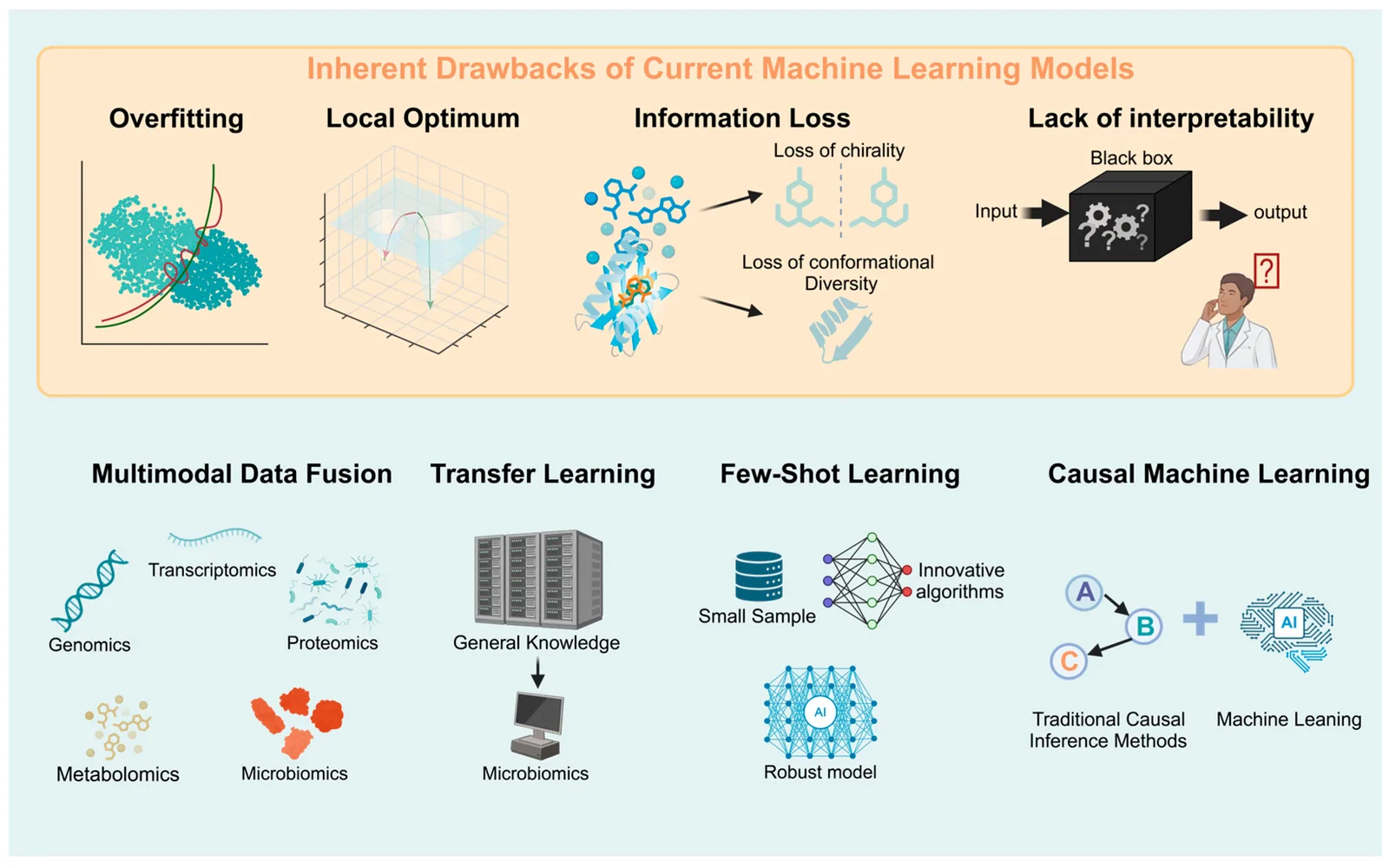
Current Limitations and Future Directions of Machine Learning. (Created in bioRender. Weihao, H. (2026) https://app.biorender.com/illustrations/69d8bc1cb106afd21f451887?slideId=9779bafe-a7d7-43d9-99ce-07ce178eab2e, accessed on 12 October 2026).

The relationships between these bottlenecks and breakthrough directions are illustrated in Figure 2. From data dependency to few-shot learning and from insufficient interpretability to “causal ML, each technical pathway corresponds to a specific practical pain point. Among these, the introduction of causal inference is particularly critical. Taking the colitis model as an example, the association between the *Lactobacillus* genus identified by Guo et al. [6] and disease status currently remains statistical. If the genetic characteristics of this bacterium were used as instrumental variables in Mendelian randomization analysis to verify its causal effect on host intestinal barrier function, the success rate of candidate probiotics in subsequent intervention experiments could be significantly improved. This exemplifies the paradigm of moving from association to causality advocated in this perspective.

Three priority directions warrant further investigation: first, introducing causal inference methods such as Mendelian randomization and causal forests into the microbial screening workflow to distinguish causal driver bacteria from companion bacteria, thereby improving the success rate of probiotic intervention development; second, developing host status prediction models that use low-cost samples such as blood as input, reducing monitoring costs and enabling dynamic nutritional intervention at the production level; third, constructing multi-center, multi-omics databases spanning different breeds and farming systems, combined with transfer learning to address model generalization under small-sample conditions (Figure 2).

## 6. Conclusions

ML in mammalian precision nutrition has covered three core areas, key microbial screening, biomarker identification, and host pathway characterization, which together form a research chain from target discovery to efficacy verification. Current challenges include model robustness under small-sample conditions, generalizability limitations due to batch effects, and insufficient interpretability of complex models. Multimodal data fusion, transfer learning, and causal inference represent breakthrough directions worthy of attention. Ultimately, translating these novel computational and multi-omics frameworks into practice holds profound promise for enhancing the overall sustainability of mammalian production systems. By precisely aligning dietary formulation with dynamic individual metabolic and microbial demands, this paradigm directly curtails redundant feed resource utilization, minimizes environmental nitrogen and phosphorus excretion, and mitigates greenhouse gas emissions. Furthermore, sustaining intestinal barrier integrity and promoting gut health through targeted bio-interventions strengthens systemic resilience and reduces reliance on prophylactic or therapeutic antimicrobials, effectively harmonizing economic efficiency, animal welfare, and ecological stewardship [57]. In the coming years, with the wider availability of low-cost multi-omics testing and the maturation of causal inference methods, ML may evolve from an auxiliary screening tool into a precision nutrition decision engine. This transformation, however, depends on a prerequisite: the research paradigm must shift from pursuing model accuracy to pursuing translatable biological discoveries, which is the core message this perspective seeks to convey.

## Figures and Tables

**Table 1:** Characteristics, biological information tiers, and translational utilities of primary omics technologies in mammalian precision nutrition.

Omics Technology	Information Provided	Key Data Characteristics & Challenges	Role in Precision Nutrition	References
Genomics	SNPs, copy number variants, Quantitative Trait Loci (QTLs)	Static, invariant over lifetime; ultra-high dimensionality; sparse.	Identifies baseline genetic potential for growth and feed efficiency; defines individual baseline requirements.	[1,3]
Transcriptomics	mRNA expression, splicing variants, spatial/cell-type patterns	Highly dynamic, tissue-specific; quantitative count matrices; susceptible to RNA degradation.	Uncovers host signaling pathways modulated by dietary transitions or additives.	[4,9,10]
Proteomics	Abundance of functional enzymes, nutrient transporters, tight junction proteins	Direct functional readout; complex dynamic range; challenging spatial resolution.	Maps mucosal barrier integrity and regional digestive/absorptive capacity (nutrient transporters) along the gastrointestinal tract.	[8,11]
Metabolomics	Concentrations of small molecules	Direct snapshot of biochemical fluxes; high chemical diversity; requires extensive compound annotation.	Supplies sensitive, circulating biomarkers for real-time monitoring of energy balance, subclinical acidosis, and nutrient utilization.	[7,12,13]
Microbiomics/Metagenomics	Taxonomic abundance (16S), microbial gene catalogs, CAZymes, metabolic pathways	High inter-individual variability; zero-inflated compositional count data; complex phylogeny.	Characterizes functional taxa fermenting fibers and protecting gut health; guides targeted probiotic, prebiotic, and postbiotic interventions.	[1,2,6,14]

**Table 2:** Summary of representative machine learning models: utilities, methodological limitations, and biological applications.

Model	Utility	Limitations	References
**Linear/Regularized Models**			
Ridge/LASSO/Elastic Net Regression	Used for feature selection of biomarkers, genes, and key microorganisms.	Incapable of learning complex non-linear feature relationships; prone to overfitting when the number of features is extremely large.	[16,17]
Support Vector Machine (SVM)	Used for protein function prediction, transmembrane protein topology prediction, disease-associated variants, and protein ordered/disordered region classification.	Difficult to scale to large-scale datasets; non-linear kernel functions reduce the intuitive interpretability of feature importance; performance evaluation is easily biased on imbalanced datasets.	[20,21,26]
k-Nearest Neighbors (kNN)	Serves as a baseline model for classification and regression; used for gene fragment classification and biomarker preliminary screening.	High inference computational overhead and poor scalability; traditional kNN uses a globally uniform fixed k that cannot adapt to local density variations; when facing irregular manifolds or data with large local structural differences, a global k cannot accommodate different regions, impairing classification performance.	[13,27]
Generalized Linear Model (GLM)	Used for feature selection of biomarkers, genes, and key microorganisms.	Linear regression models generally perform suboptimally on classification tasks; direct application to high-dimensional raw features yields relatively poor performance.	[28,29,30]
**Tree-based Models**			
Random Forest (RF)	Used for predicting disease-associated genomic mutations, protein–ligand interactions, colorectal cancer classification, and growth rate estimation; also applied to molecular structure and expression prediction.	Relatively less suited for regression tasks; aggregating large numbers of decision trees makes overall interpretation difficult; inconsistent cross-task performance.	[23,24]
Gradient Boosting (e.g., XGBoost)	Used for gene expression profiling, drug activity prediction and classification, and cancer prediction.	Capable of learning feature importance and insensitive to feature scaling, but remains prone to noise.	[31,32,33]
**Deep Learning**			
Convolutional Neural Networks (1D/2D/3D CNN)	Protein sequence analysis, interaction prediction, biomolecular spectroscopy, and virtual drug screening.	Relies on supervised learning and requires large amounts of human-annotated data; imperceptible perturbations added to input images can cause CNNs to output high-confidence misclassifications.	[34,35,36]
Recurrent Neural Networks (RNN)	Used for genomic and non-coding RNA sequence analysis, protein sequence analysis, and cheminformatics/bioinformatics.	Incapable of parallel computation; poor black-box interpretability; sensitive to noise and prone to overfitting; performance degrades further when annotated sequence data in biology is scarce.	[37,38,39,40]
Graph Neural Networks (GNN)	Used for virtual drug screening, biomarker discovery, protein structure analysis, and biological network analysis.	High memory footprint and computational cost; highly sensitive to graph noise and missing edges (common issues in biological networks); weak interpretability.	[41,42,43,44]
Long Short-Term Memory (LSTM)	Overcomes the vanishing and exploding gradient problems inherent in conventional Recurrent Neural Networks (RNNs) by leveraging gated architectures (input, forget, and output gates) to retain information across extended temporal horizons.	Inability to Support Fully Parallel Computation and High Computational Cost.	[45]
**Causal Machine Learning**			
Causal ML (e.g., Causal Forest, DoubleML)	Unlike traditional predictive machine learning that only evaluates correlations or predicts risk scores, causal machine learning quantifies outcome changes caused by specific interventions (such as drug treatments), enabling individualized and heterogeneous treatment effect estimation.	Highly dependent on strong predictive variables and large sample sizes; most models lack rigorous uncertainty quantification.	[46,47,48,49,50]
**Transfer Learning**			
Transfer Learning	Substantially reduces reliance on labeled target-task data; enables cross-omics and cross-modal knowledge transfer.	Prone to amplifying spurious correlations and leading to overfitting.	[51,52,53]

## Data Availability

No new data were created or analyzed in this study. Data sharing is not applicable to this article.

## Abbreviations

The following abbreviations are used in this manuscript:

ML	Machine learning
LASSO	Least absolute shrinkage and selection operator
RF	Random forest
SVM	Support Vector Machine
XGBoost	Extreme Gradient Boosting
kNN	k-Nearest Neighbors
GLM	Generalized Linear Model
CNN	Convolutional Neural Networks
RNN	Recurrent Neural Networks
GNN	Graph Neural Networks
